# Heavy Metal Accumulation in Dominant Green Algae Living in a Habitat Under the Influence of Cu Mine Discharge Water

**DOI:** 10.3390/plants14070993

**Published:** 2025-03-21

**Authors:** Merve Sasmaz Kislioglu, Erdal Obek, Nevin Konakci, Ahmet Sasmaz

**Affiliations:** 1 Department of Environmental Engineering, Firat University, Elazığ 23119, Turkey; msasmaz91@hotmail.com; 2 Department of Bioengineering, Firat University, Elazığ 23119, Turkey; eobek@firat.edu.tr; 3 Department of Geological Engineering, Firat University, Elazığ 23119, Turkey; nevinozturk@firat.edu.tr

**Keywords:** *Ulothrix* sp., mine drainage, heavy metals, algae, bioremediation, environment

## Abstract

Algae absorb nutrients such as nitrogen (N) and phosphorus (P), as well as dissolved metal ions from polluted waters, and accumulate them in their tissues, thus contributing to the decontamination of water. This feature enables them to be used both as bioindicators of water pollution and in bioremediation applications aimed at the remediation of these waters. This study aims to define the dominant macroscopic green algae species developing in habitats affected by acidic leaks and currents from the copper mine operation site located in the Maden district of Elazığ province (Türkiye) and determine the extent to which these algal biomasses bioaccumulate selected metals (As, Cu, Cr, Hg, Cd, Ni, Pb, Zn) and contribute to water decontamination. For these purposes, metal (Fe, Cu, Zn, Mn, Cr, Pb and Cd) analyses were conducted on the algal biomasses collected from the abovementioned habitats and on water samples using Inductively Coupled Plasma-Mass Spectrometry (ICP-MS). The dominant algal biomasses collected from the seepage water and Maden River habitats were identified as *Ulothrix variabilis Kuetzing* and *Ulothrix tenuissima Kützing*, respectively. Heavy metal concentrations (mg/kg dry weight) in the biomasses of *Ulothrix variabilis Kuetzing* and *Ulothrix tenuissima Kützing* species were determined as follows: Fe (11,094 mg/kg; 8.26 mg/kg) > Cu (6787 mg/kg; 180 mg/kg) > Zn (680 mg/kg; 283 mg/kg) > Mn (525 mg/kg; 13 mg/kg) > Co (838 mg/kg; 64 mg/kg) > Ni (472 mg/kg; 95 mg/kg)> Cr (164 mg/kg; 107 mg/kg) > Pb (83.6 mg/kg; 68.7 mg/kg) > Cd (1.48 mg/kg; 5.40 mg/kg), respectively. It was found that the affinity of both algal biomasses for the selected metal ions decreased in the order of Fe > Cu > Zn > Mn > Cr > Pb > Cd. Also, according to the calculated bioconcentration factor, it was shown that both algal biomasses were very good heavy metal accumulators. As a result, both algal biomasses can be used as effective biomonitoring agents and bioremediators for acidic and metal-laden polluted waters.

## 1. Introduction

Although 71% of the Earth is covered with water, approximately 3% of this is fresh water. Approximately 13% of fresh water is found on the surface (streams, rivers, lakes, etc.). Fresh water, which is invaluable for human, animal and plant life as well as vital for agricultural and industrial purposes, is supplied from surface and underground resources, while countries with limited water resources meet their water needs via the seas. Although the purification of salt water is a process that involves expensive and laborious processes in terms of cost, it still cannot achieve the same quality as fresh water [1,2].

Numerous chemical and physical remediation studies have been conducted to attempt to control the negative environmental effects of mine drainage waters. These vary from cheap technologies such as microbial phytoremediation and remediation to expensive technologies such as ion exchange, chemical precipitation, reverse osmosis and electrodialysis [3]. Phytoremediation is a method in which microorganisms such as algae, fungi and bacteria are used to accumulate heavy metals from both water and soil [4]. The use of microorganisms such as yeast, fungi, bacteria, and algae in phytoremediation processes is also known as biosorption for both non-living and living biomasses [5,6,7,8,9], and phytosorption, as well as phytoremediation, in the case of the accumulation of heavy metals from aquatic environments using only algae [10]. In some cases, the absorption and adsorption of heavy metals by organisms is simply referred to as bioaccumulation [5], but if there is enough heavy metal absorption, it can cause some toxic symptoms to develop in plants [11,12]. Some researchers in recent years have studied the ability of algae to adsorb and absorb heavy metals, phosphorus and nitrates from aquatic environments [13]. Various algae species have been detected growing in aquatic areas contaminated with heavy metal ions. For example, sweet filamentous green algae, *Stigeoclonium* sp. was observed to grow in mining water polluted with high heavy metal concentrations and exhibited a relatively high accumulation ability [13]. Similarly, *Mougeotia* sp., another filamentous green alga present in the southeastern part of Western Australia, was observed with a “red rusty” color in the acidic mining waters of coal mines [14]. It was found that this plant has the capacity to remove high values of Al and Fe in a pH range between 3 and 6 [14]. Adsorption of heavy metals in aquatic environments generally forms on the cell wall and by binding to other intracellular molecules, metallothioneins, phytoclatins and cytoplasmic ligands [15]. The cell surface of green algae contains hetero-polysaccharides with sulfate groups and carboxyl, which aid in the removal of heavy metal concentrations [5]. Additionally, some researchers have reported that metals such as Ni, Zn, Cd, Hg, Sr, Co, Mg, Pb, Cu and Ti are retained in the polyphosphate parts of green algae. Algae form a “detoxification mechanism” and “storage pool” for heavy metals [16]. The accumulation of heavy metals from aquatic environments by algae occurs through two processes: passive uptake and active uptake [5,9]. Biodiversity dominated by acid-tolerant and acidophilic organisms is heavily affected by low pH, dissolved metal ions and high sulfate concentrations [17]. Despite the varying chemical and physical conditions in contaminated environments, some algae species can adapt to these environments and form large communities. *Klebsormidium* sp., *Eunotia exigua*, *Mougeotia* sp., *Pinnularia acoricola* Hust, *Zygnema* sp., *Ulothrix* sp. and *Euglena mutabilis* Schmiditz are frequently observed in environments with acidic mineral waters [18].

The study area and its surroundings are rich in sulfide mineralization; acidic mine waters are formed in these areas, and these contaminated waters cause pollution in the Tigris basin. This study aims to investigate the metal accumulation and water decontamination capacity of green macroalgae (*Ulothrix variabilis* Kuetzing and *Ulothrix tenuissima* Kützing) growing in the operating discharge waters of the Maden copper deposit (Elazig, Türkiye), located at the source of this basin.

## 2. Materials and Methods

### 2.1. Sampling Site

**Figure 1:**
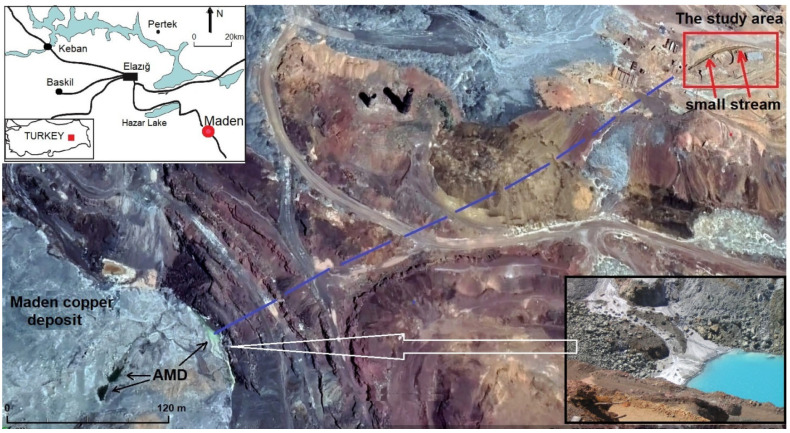
Location map of the study area (taken from Sasmaz Kislioglu [21] and Konakci [26]).

The Maden Cu deposit has been mined for copper by different civilizations for about 4000 years (Figure 1). The ore body has a bowl-shaped geometry and is approximately 0.5 km wide, 1 km long and 0.17 km deep. The Maden Cu deposit is a volcano-sedimentary Cyprus-type copper deposit, mainly composed of pyrite, arsenopyrite, chalcopyrite, magnetite, bornite, pyrrhotite, calcite, quartz, barite, calcite, and secondary limonite, chalcocite and covellite. The area is heavily polluted by both natural ore deposits and ancient mining operations.

The climate in the region is subtropical, with an average of 554 mm of precipitation throughout the year. The annual average temperature is 19 °C, and monthly average temperatures vary between 15 °C in winter and 24 °C in summer.

### 2.2. Water and Algae Sample Collection

**Figure 2:**
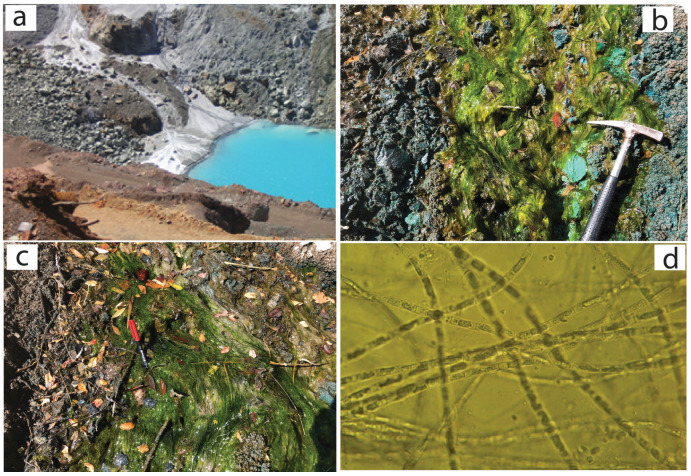
(**a**) Maden Cu mining area, (**b**,**c**) *U. variabilis* Kuetzing, (**d**) *U. variabilis* Kuetzing image under a microscope.

**Figure 3:**
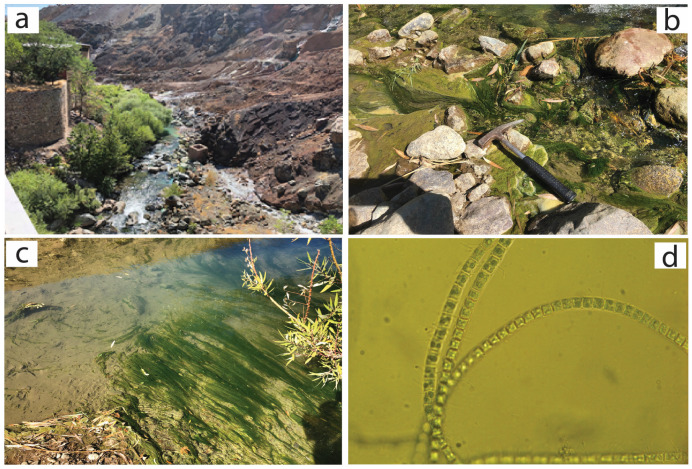
(**a**) Maden River, (**b**,**c**) *Ulothrix tenuissima* Kützing, (**d**) *Ulothrix tenuissima* Kützing image under a microscope.

Water and plant sampling procedures were carried out in August 2021. Mineral water, with a high metal content, was collected from both surface and underground sources in the abandoned mining production and ore area (Figure 2). Because the facility is no longer in operation, mine drainage water maintains a consistent composition throughout the year [19,20,21]. *Ulothrix variabilis* Kuetzing algae is observed in the study area, typically in areas where mineral water flows vigorously, and it is a naturally occurring plant that exists year-round. The *Ulothrix tenuissima* Kützing plant, on the other hand, is commonly observed only in ponds where the water is more stagnant in the Maden River (Figure 3). These plants begin to grow in the spring, with the fastest growth occurring from July to September. Plant growth halts between October and December.

The green algae were collected from the mining water of the Maden Cu deposit and the Maden River. The coordinates of the mining water site are 38.388670° N and 39.678936° E, and the coordinates of the Maden River sample site are 38.390650° N and 39.677479° E.

Algae samples were collected from an area approximately 2 m long and 50 cm wide in the leachate area and samples were placed in high-density polyethylene plastic bottles and preserved with Lugol for identification. Optical microscopy studies based on taxonomic identification, morphological features and color tests of the algae samples were carried out by Prof. Dr. Bülent Sen and Prof. Dr. Feray Sönmez, as described by Bicudo and Menezes [22].

### 2.3. Physical and Chemical Analysis of Water Samples

Two different water samples were collected in the field. The first sample was taken from the water coming from the Maden Cu deposit, and the other water sample was taken from the Maden River. Both water samples were collected in plastic bottles to investigate physicochemical parameters and perform anion–cation analyses. Additionally, the pH, electrical conductivity (EC) and temperature of the samples were measured on site. Then, samples were transported to Acme Laboratory (Vancouver, BC, Canada) in sterilized and clean plastic bottles for analysis. The physical and chemical parameters of the water samples were measured at the DSI laboratory (Elazig, Türkiye), which is accredited by ISO 17025. Then, pH, EC (electrical conductivity), color, turbidity, conductivity, acidity, various metals, sulfates, total Kjeldahl N (TKN) and phosphorus were analyzed in both water samples.

### 2.4. Algae Sample Preparation and Bioconcentration Factor (BCF) Determination

Algae samples from the study area were carefully collected, as seen in Figure 2b,c and Figure 3b,c, and then transported to the laboratory. The green algae samples were washed first, then dried at 60 °C, weighed, and then washed at 300 °C for 24 h. Finally, they were digested using a mixture of HNO_3_, HCl, and H_2_O (1:1:1, *v*/*v*; 6 mL per 1.0 g of sample) for one hour and then analyzed by ICP-MS for different elements. After the ICP-MS analysis of the green algae, all results were converted to dry weight. All metal analyses were evaluated in a certified laboratory accredited for calibration and testing competency according to the ISO/IEC 17025 general requirements. The analyses were performed in an ICP-MS Perkin Elmer Elan 9000 using the certified reference material V16 for plants [21]. Repeated analyses showed better than 5% reproducibility.

The bioconcentration factor (BCF) is calculated by dividing the amount of metal in the algal biomass by the metal concentration in the water in which it grows and is expressed as a percentage (%) [23,24,25]. This factor was calculated using the formula [BCF = metal concentration in algae (mg/kg)/metal concentration in water (mg/L)].

All data were tested by using SAS 9.4 (SAS Institute, Cary, NC, USA) for the ANOVA variance analyses with a critical *p*-value of 0.05.

## 3. Results and Discussion

The studied water samples were taken from both Maden River and the area where the copper deposit is located. The physicochemical parameters, anion and cation analyses and trace element concentrations of these waters are given in Table 1. According to these results, while Maden River water has a pH value of 8.42, indicating a basic character, mining water has a pH value of 5.82, indicating an acidic character. In addition, mining water has high EC, SO_4_, Cl^−^, F^−^ and total N concentrations when compared to the water from Maden River. The high SO_4_ content in mining water coming from the Cu deposit can be related to the high pH of this water and common sulfide minerals in this deposit, including pyrite (FeS_2_), arsenopyrite (FeAsS), sphalerite (ZnS), chalcopyrite (CuFeS_2_) and galena (PbS).

Although the Maden River water has lower metal concentrations compared to the mining water from the copper deposit, *Ulothrix tenuissima* Kützing accumulated metals at significantly higher levels than those present in the water. For example, the accumulation was 1.625 times higher for Ag, 61 times higher for As, 32 times for Cd, 64 times for Co, 750 times for Cu, 405 times for La, 942 times for Mn, 792 times for Ni, 382 times for Pb, 19 times for Sb, 7.5 times for Th, 5.3 times for Tl, 11 times for U, 14 times for V, 7 times for W, and 3.6 times for Zn. Similarly, *Ulothrix variabilis* Kuetzing accumulated 163 times for Ag, 430 times for Au, 53 times for Hg, 2.0 for W and 1.04 times for Ni from mining water. These results indicate that both *U. tenuissima* and *U. variabilis* plants have the ability to accumulate metals in water at high rates. Especially if the water in which the plant lives has both a high metal concentration and an acidic character, the plant tends to accumulate metals easily from such water. If the water has a high metal concentration and a basic pH, the plant is reluctant to accumulate metals in the water.

Trace element concentrations of *Ulothrix variabilis* Kuetzing and mining water are given in Table 1. When the trace element results of the mining water are examined, it is evident that the element concentrations are much higher than those in the Maden river water. This situation can be observed when the EC values of both waters are examined: the EC values of the mining water are much higher than those of the river water (Table 1). Similarly, the trace element concentrations of *Ulothrix variabilis* are higher than the trace element concentrations of *U. tenuissima*; therefore, it can be observed that *Ulothrix* variabilis accumulates more trace elements from the water than *U. tenuissima*. When the BCF values of *Ulothrix variabilis* are examined, it can be seen that it has much lower BCF values than *U. tenuissima*. *U. variabilis* accumulated metals from the water at a high rate due to the high heavy metal concentration in the mine water. Similarly, *U. tenuissima* also accumulated heavy metals observed at lower concentrations in the Maden River water at a high rate and had high BCF values. As a result, *U. variabilis* has high accumulation performance and *U. tenuissima* has high BCF values.

The removal of metal by algae is highly variable, depending on age of plant, the taxon, and the metal and physicochemical parameters of the environments [27]. Optimum metal accumulation and removal also vary with season [28]. Algae release extracellular polysaccharides, especially under nutrient stress conditions [29,30]. Different reports have demonstrated that organisms able to produce extracellular polysaccharides exhibit metal accumulation abilities [31].

Haritonidis and Malea [32] studied the accumulation of Fe, Cd, Cu, Zn and Pb in seawater and sediment at five stations in Hermaikos Bay, which was polluted by industrial and domestic waste, using the green alga *Ulva rigida*. The metal levels in the algae were distributed equally among the five stations, and high positive correlations were observed among Pb-Cu, Fe-Cu and Cd-Pb in *U. rigida*. *U. rigida* accumulated high levels of Pb, Zn and Cd, similar to green algae in the study area. Therefore, *U. rigida* can be considered as an indicator plant for Cd, Pb and Zn accumulation due to its high accumulation ability.

The bioconcentration factors of the studied algae samples for different metals are given in Table 1. The results show that both *U. tenuissima* and *U. variabilis* bioaccumulate metals across a wide and varying range. For example, *U. tenuissima* accumulated metals at a high rate in Maden River with low metal concentration, and the BCF sequence here was Ag > Mn > Ni > Cu > La > Pb > Co > As > Sb. Therefore, Ag, Mn, Ni and Cu have the highest bioaccumulation rates in the study and these results are in accordance with the studies of Akcali and Kucuksezgin [33] and Du et al. [34]. Although zinc is also a biologically essential element, its bioaccumulation factor is quite low compared to other metals and was calculated as 3.6. *U. variabilis* has low BCF rates due to the high metal concentration in Maden mine water. The metal sequences here are Au > Ag > Hg > Cu > W > Ni from highest to lowest. The other analyzed metals had BCF values below one.

The function of native algae in removing metals from acid mine drainage in Frongoch Mine (UK) was examined by Du et al. [34]. Acid mine drainage (AMD) samples were gathered in June and October 2021 in order to assess the values of Fe, Zn, Cu, Pb, Cd, and As in the algae. High concentrations of Pb (4.22 mg/L) and Zn (351 mg/L) in AMD samples surpassed water quality limits, and their pH values ranged from 3.5 to 6.9. The two primary species of algae in the Frongoch AMDs were *Ulothrix* sp. and *Oedogonium* sp. The bioconcentration factor of metals followed a descending order of Fe > Pb > Cu > Cd > Zn, with metal concentrations in algae ranging from 0.007 mg/kg to 51 mg/kg. It was discovered that Pb and Fe were primarily bioaccumulated in the algae, whereas Zn, Cu, and Cd were adsorbed onto their surface and bioaccumulated in the algae. Native algae can be utilized in bioremediation techniques and can be thought of as a biogeochemical barrier where metals are building up. Additionally, Frongoch Mine and others of a similar nature could use native algae as a bioindicator to evaluate water contamination.

According to Massocato et al. [35], one of the primary factors contributing to the deterioration of soil and water resources is the formation of AMD. Heavy metals may be bioaccumulated by organisms found at AMD-contaminated sites, which would encourage their use in bioremediation procedures. *Ulothrix* sp. was found among the algal species isolated from AMD-contaminated water in the Sideropólis region of Brazil. In relation to zinc, manganese, and nickel, the current study assessed its tolerance and bioaccumulation potential. The effects of varying Zn, Mn, and Ni concentrations—both separately and in combination—on the alga’s physiological function were examined through experiments. Only the cells exposed to doses greater than 0.55 mM Zn exhibited a reduction in growth rate and harm to physiological functions, according to the data. The findings indicate that, at least at the pH that was assessed, the dispersion of this alga in polluted medium is unaffected by the content of Ni and Mn. According to our findings, *Ulothrix* sp. can withstand and thrive in environments with higher metal concentrations than have been previously documented for AMD.

Malakootian et al. [36] examined the biosorption of copper, zinc, lead, and cadmium from industrial wastewaters by *Ulothrix zonata* algae. *Ulothrix Zonata’s* removal efficiency was examined in relation to algal dosage, initial heavy metal concentration, sorption time, particle size, pH, and temperature. First and second order kinetics have been applied to the analysis of the experimental data. Copper, zinc, lead, and cadmium were removed at levels of 98.2%, 96%, 98.4%, and 94.7%, respectively, under conditions of 25 °C, pH 4–5, a contact period of 60 min, and an adsorbence level of 1.5 g/L. Among the variables examined here, pH was crucial in determining the metals’ absorption level; the highest absorption level was found at pH values between 3 and 5. According to isothermal characteristics, adsorbing lead has a greater adsorbent capacity, whereas adsorbing cadmium requires more energy. *Ulothrix zonata* algae have high absorption efficiency, making them suitable for the biosorption of heavy metals from industrial wastewater.

Saavedra et al. [37] conducted a comparative uptake study of four green microalgae species (*Chlorophyceae* sp., *Chlamydomonas*, *Chlorella vulgaris* and *Scenedesmus almeriensis reinhardtii*) in monometallic and multimetallic solutions, assessing the impact of contact time and pH. The highest removal efficiency values for each toxic element were 38.6% for B (*S. almeriensis*, pH 5.5, 10 min), 40.7% for As (*S. vulgaris*, pH 9.5, 3 h), 91.9% for Zn (*Chlorophyceae* sp., pH 5.5, 3 h), 88% for Cu (*Chlorophyceae* sp., pH 7.0, 10 min), and 99.4% for Mn (*C. vulgaris*, pH 7.0, 3 h). B removal efficiency dropped significantly in multimetallic solutions (down to 0.2% in *C. reinhardtii*), with the exception of the single species isolated from a contaminated environment, *Chlorophyceae* sp. FTIR spectra revealed that Cu (3300, 1741, 1535, 1350–1400 cm^−1^) and As (1150–1300 cm^−1^) had the strongest interactions. The results validated the potential of microalgae biomass as a biosorbent for harmful substances.

According to Pham et al. [38], microgreen algae have a great potential for both producing biodiesel and removing heavy metals from wastewater. This study investigated the uptake of metals and the buildup of lipids using the green algal species *Scenesesmus* sp. In a laboratory-scale system, the microalga *Scenedesmus* sp. was cultivated continuously while exposed to Pb at concentrations of 0.05, 0.5, 1, 2, and 10 mg/L. With a 96 h EC50 of 4.76 mg/L, the results showed that Pb suppressed algal growth. At low concentrations, Pb could be effectively removed by the green algae Scenedesmus sp. Pb addition at up to 1 mg/L resulted in a considerable increase in lipid accumulation of up to 31%. The treatment with the greatest Pb concentrations (10 mg/L), on the other hand, showed reduced lipid accumulation and poorer heavy metal removal efficiency. Based on the results, Pb concentrations above 2 mg/L restrict algal development, which in turn decreases the buildup of lipid content in the cell. According to this study, the green algae Scenedesmus sp. can extract lead from aqueous media and build up lipid content, which may be useful in the manufacture of biodiesel and wastewater treatment technologies.

According to Li et al. [39], *Chlorella* and *Scenedesmus* species can extract up to 89% of lead from aqueous solutions. However, factors including initial concentration, exposure time, and target species affect how quickly microalgae remove metal ions. Algae can absorb metals passively or actively. While some metals, like Pb and strontium (Sr), are actively absorbed against a large intracellular concentration gradient, others, like Zn and Cd, may be passively adsorbed by charged polysaccharides in the intracellular matrix and cell wall. Chen et al. [40] used a feedback mechanism involving multiple transporters, together with the presence of certain cations or other metal ions like copper (Cu) and Ni, to explain their observations of increasing Pb bioaccumulation in the green alga *C. reinhardtii*. Algae usually absorb metal primarily through adsorption onto the cell surface and (a slower) active absorption into the cytoplasm [41]. Nonetheless, Flouty and Estephane [42] discovered that in binary metal systems, Cu and Pb had both antagonistic and synergistic effects, suggesting that the bioaccumulation process is far more dynamic. It is believed that the Ca^2+^ pathway is responsible for Pb^2+^ uptake in green algae.

*Scenedesmus* sp. was shown by Flouty and Khalaf [43] to be capable of effectively absorbing and eliminating lead from aqueous solutions at low concentrations. Increased Pb concentrations hampered cell development, which in turn reduced the elimination capability. The current study’s findings support earlier findings that *Scenedesmus* sp. can accumulate metals to a certain degree, contingent on the metal’s concentration and the length of time the algae are in contact with it.

## 4. Conclusions

The water basin around the copper mine in the Maden district of Elazığ is heavily affected by leakage and discharge water, causing heavy pollution of clean water resources, especially along the lower Maden River basin. The results of this study clearly reveal the negative effects of AMD on algae living in the aquatic environment of the region. Metal accumulations in basic (pH 8.42) Maden River water with *Ulothrix tenuissima* Kützing, which is dominantly observed in the region, and the heavy metal accumulation capacities in acidic mine water (pH 5.82) with filamentous green macroalgae *Ulothrix variabilis* Kuetzing were investigated. Accordingly, the BCF values for *Ulothrix tenuissima* Kützing were found to be 1,625 for Ag, 942 for Mn, 792 for Ni, 750 for Cu, 405 for La, 382 for Pb, 64 for Co, 35 for Ti, 11 for U, 8 for Th, 14 for V, 3.6 for Zn, and 19 for Sb. This shows that the *U. tenuissima* plant accumulates the above metals in the river at a high rate. The *U. variabilis* plant has taken up the metals in the mine water containing high metal levels to a significant extent, but among various metals, the BCF values for this plant were only calculated as 163 for Ag, 1.91 for Cu, 1.04 for Ni, and the BCF values for other metals were generally found to be lower than one. *U. variabilis* accumulated metals from the water at a high rate due to the high heavy metal concentration in the mine water. Similarly, *U. tenuissima* also accumulated heavy metals observed at lower concentrations in the Maden River water at a high rate and had high BCF values. As a result, *U. variabilis* has high accumulation concentrations and *U. tenuissima* has high BCF values. The results of both algae plants showed that they have the ability to accumulate heavy metals at a high rate both in waters with low heavy metal concentrations and in acidic AMD waters with high heavy metal content. This indicates that both plants will be highly effective in cleaning and remediating heavy metals in such polluted areas.

## Figures and Tables

**Table 1:** Physicochemical characteristics of Maden river water and Maden mining water and their anion–cation analysis results for *U. tenuissima* and *U. variabilis* plants.

	Det. Limit (ppb)	*Maden River* (ppb)	*U. tenuissima* (n = 5 Samples) (mg/kg)	BCF	Mining Water (ppb)	*U. variabilis* (n = 6 Samples) (mg/kg)	BCF
T °C	-	18.6 ± 1.2	-	-	19.3 ± 1.4	-	-
pH	-	8.42 ± 0.6	-	-	5.82 ± 0.4	-	-
EC mS cm^−1^	-	1.55 ± 0.02	-	-	2.49 ± 0.03	-	-
HCO_3_ mg L^−1^	-	345 ± 15	-	-	288 ± 18	-	-
NO_3_^−^ mg L^−1^	-	2.18 ± 0.11	-	-	1.88 ± 0.02	-	-
SO_4_ mg L^−1^	-	61.7 ± 4	-	-	122 ± 8	-	-
Cl^−^ mg L^−1^	-	4.88 ± 0.3	-	-	6.54 ± 0.5	-	-
F^−^ mg L^−1^	-	0.26 ± 0.02	-	-	0.38 ± 0.02	-	-
Total N	-	0.62 ± 0.05	-	-	0.76 ± 0.03	-	-
Kjeldahl N	-	0.18 ± 0.01	-	-	0.23 ± 0.01	-	-
Ag (ppb)	0.05	0.08 ± 0.01	130 ± 8	1625	9.25 ± 0.7	1509 ± 78	163
Al	1	106 ± 8	1.62 ± 0.2	0.02	24045 ± 36	0.47 ± 0.01	0.01
As	0.5	0.7 ± 0.01	42.5 ± 2	61	170 ± 10	67.3 ± 0.4	0.40
Au (ppb)	0.05	<0.05	55.4 ± 4	-	0.7 ± 0.02	301 ± 22	430
B	5	32 ± 2.1	7 ± 0.5	−0.22	840 ± 24	21 ± 1.8	0.03
Ba	0.05	11.4 ± 0.8	60 ± 5	5.26	450 ± 22	53.9 ± 4.2	0.12
Cd	0.05	0.17 ± 0.02	5.4 ± 0.4	32	6.2 ± 0.04	1.48 ± 0.12	0.24
Co	0.02	1.4 ± 0.01	64 ± 3	64	1725 ± 52	838 ± 55	0.49
Cr	0.5	76.6 ± 4.3	107 ± 11	1.40	200 ± 14	164 ± 12	0.82
Cu	0.1	0.24 ± 0.02	180 ± 15	750	3562 ± 66	6787 ± 128	1.91
Fe	10	370 ± 22	8.26 ± 0.8	0.02	115750 ± 87	11094 ± 48	0.10
Hg	0.01	<0.01	85 ± 6	-	1.8 ± 0.02	96 ± 7.2	53
La	0.01	0.02 ± 0.01	8.09 ± 0.9	405	9.45 ± 0.6	1.01 ± 0.06	0.11
Li	0.05	<0.05	9.27 ± 0.8	-	23 ± 1.5	4.54 ± 0.3	0.20
Mn	0.05	0.14 ± 0.01	13 ± 1.4	942	6460 ± 106	525 ± 38	0.08
Mo	0.01	<0.01	6.4 ± 0.5	-	30 ± 2.8	10.5 ± 0.8	0.35
Ni	0.02	0.12 ± 0.01	95 ± 7	792	455 ± 28	472 ± 23	1.04
Pb	0.02	0.18 ± 0.01	68.7 ± 6	382	285 ± 15	84 ± 7	0.29
Sb	0.05	0.11 ± 0.01	2.09 ± 0.3	19	18.5 ± 1.2	2.87 ± 0.3	0.16
Sr	0.01	163 ± 12	174 ± 13	1.02	2585 ± 75	26.2 ± 1.8	0.01
Th	0.05	0.08±0.01	0.6 ± 0.01	7.5	0.48 ± 0.03	0.2 ± 0.01	0.42
Ti	10	12 ± 1.3	420 ± 33	35	186 ± 14	101 ± 8.4	0.54
Tl	0.01	0.06 ± 0.01	0.32 ± 0.01	5.33	6.04 ± 0.5	0.37 ± 0.02	0.06
U	0.02	0.13 ± 0.01	1.4 ± 0.01	11	4.2 ± 0.2	0.11 ± 0.01	0.03
V	0.02	1.7 ± 0.02	23 ± 2.4	14	96.4 ± 9	91 ± 6.4	0.94
W	0.02	0.03 ± 0.01	0.2 ± 0.01	7	0.10 ± 0.01	0.2 ± 0.01	2.00
Zn	0.5	78.9 ± 12	283 ± 14	3.6	2850 ± 36	680 ± 44	0.24

## Data Availability

Data are contained within the article.
